# Geraniol as a Potential Stimulant for Improving Anthocyanin Accumulation in Grape Berry Skin through ABA Membrane Transport

**DOI:** 10.3390/plants11131694

**Published:** 2022-06-27

**Authors:** Norika Mikami, Mayu Konya, Shinichi Enoki, Shunji Suzuki

**Affiliations:** Laboratory of Fruit Genetic Engineering, The Institute of Enology and Viticulture, University of Yamanashi, Yamanashi 400-0005, Japan; g22lf016@yamanashi.ac.jp (N.M.); tomomisuzuki.ss@gmail.com (M.K.); senoki@yamanashi.ac.jp (S.E.)

**Keywords:** abscisic acid, ABA membrane transport, anthocyanin, geraniol, grapevine

## Abstract

Climate change, particularly warmer temperature, has resulted in reduced anthocyanin accumulation in grape berry skin. Because anthocyanin is a crucial determinant of red wine quality, viticulturists need to devise a solution for mitigating the poor coloration of red/black grape berry skin under elevated temperature conditions. In this study, we investigated the effects of geraniol on anthocyanin accumulation in grape berry skins of field-grown grapevines and elucidated the molecular mechanisms of the geraniol-triggered anthocyanin accumulation. Geraniol-treated bunches showed enhanced anthocyanin accumulation in berry skins at harvest (50 days after treatment). Geraniol treatment upregulated the transcription of *MybA1* and *UFGT*, which encode the key factors in anthocyanin biosynthesis, in berry skins. Geraniol treatment also improved anthocyanin accumulation in grape cultured cells. We isolated grape ATP-binding cassette transporter G family protein *VvABCG40*, encoding abscisic acid (ABA) membrane transporter, from geraniol-treated grape cultured cells. *VvABCG40* transcription was upregulated in berry skins 40 days after treatment. Geraniol treatment also upregulated the transcription of *VvPP2C24*, which encodes ABA-responsible type 2C protein phosphatases, in berry skins, but not the transcription of *VvNCED1*, which encodes a key enzyme in ABA biosynthesis. Taken together, geraniol-triggered anthocyanin accumulation in berry skins is promoted by ABA membrane transport and not by ABA biosynthesis, and geraniol treatment of field-grown grape bunches may contribute to alleviating the poor coloration of berry skin as a novel technique in viticulture.

## 1. Introduction

Anthocyanin content in grape berry skin is one of the determinants of red wine quality [1]. Anthocyanin is synthesized from phenylalanine through the phenylpropanoid, flavonoid, and anthocyanin biosynthetic pathways in grape berry skin [2]. Anthocyanin content in grape berry skin is generally affected by environmental conditions [3]. For example, anthocyanin biosynthesis in grape berry skin was inhibited under high nighttime temperature because of the low expression of anthocyanin-biosynthesis-related genes [4]. The rapid increase in global temperatures has resulted in the poor coloration of grape berry skin in current wine grape-producing areas and initiated the shift of viticulture regions northward or to higher altitudes [5]. Viticulturists also face the issue of having to decide whether traditional red wine cultivars should be changed to high-temperature-tolerant cultivars. Cultivar diversity may mitigate potential losses in grape-producing areas in the age of global warming [6].

Viticultural practices, including trunk girdling [7], leaf removal [8], and cluster thinning [9], have been developed to mitigate the effects of global warming on grape berry skin coloration [10]. It was recently reported that sound stimulation (sine wave sound at 1000 Hz) enhanced anthocyanin accumulation in grape berry skins of field-grown grapevines at the early stage of ripening [11]. However, these cultivation techniques are expensive and require expertise and a heavy workload. Another strategy to maintain the coloration of grape berry skin is the application of chemical stimulants to grape bunches. The application of chemical stimulants is simpler than the viticultural practices described above. Abscisic acid (ABA) regulates anthocyanin biosynthesis in grape berry skin [12]. The application of ABA to grape bunches enhanced anthocyanin accumulation in berry skin [13]. In addition, the increase of endogenous ABA level in grape berry skin triggered the coloration of berry skin during berry maturation [14]. Vanillylacetone, a pungent component of ginger, upregulated the expression of anthocyanin-biosynthesis-related genes responsible for anthocyanin accumulation by enhancing endogenous ABA biosynthesis [15]. Allantoin, an intermediate metabolite in plant purine catabolism, enhanced anthocyanin accumulation in grape berry skins by releasing bioactive ABA from glucose-conjugated ABA [16]. Thus, the application of chemical stimulants enhancing ABA biosynthesis and/or accumulation is an alternative strategy to maintain the coloration of grape berry skin under elevated temperature conditions.

The objective of this study was to evaluate the use of a volatile compound, geraniol, as a potential stimulant for improving anthocyanin accumulation in the grape berry skins of field-grown grapevines. Volatile compounds emitted by plants mediate the interaction of plants with insects, microorganisms, and other plants [17]. Six-carbon aldehydes, alcohols, and esters emitted by corn seedlings in response to damage by herbivores primed neighboring seedlings against impending herbivores by enhancing their chemical defenses [18]. 6-Pentyl-2H-pyran-2-one emitted by *Trichoderma* spp. activated the plant defense response in grapevines [19]. Resistant genotypes of grapevines produced 2-ethylfuran, 2-phenylethanol, β-cyclocitral, or trans-2-pentenal, which contributed to the defense mechanisms of resistant grapevines [20]. Although the role of volatile compounds in plant–plant signaling and plant defense responses has been characterized, the contribution of volatile compounds to grape berry skin coloration has not yet been investigated.

**Figure 1:**
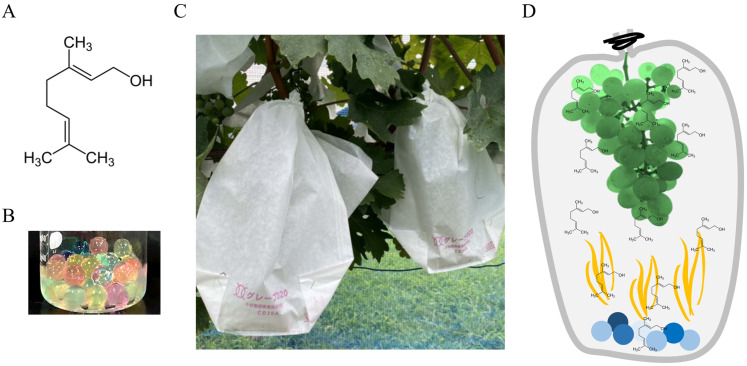
Experimental design of geraniol treatment to bunches of field-grown grapevines. (**A**) Chemical structure of geraniol. (**B**) Water-absorbing polymer treated with 10 nM geraniol. Polymer treated with diluted ethanol was used as control. (**C**) Photograph of geraniol-treated bunches of field-grown grapevines. Geraniol-treated or control polymer was added into a grape protective bag, respectively. The bunches were covered with the bags at véraison. Bunches were randomly collected every 10 days from véraison to harvest. (**D**) Schematic representation of geraniol treatment to bunches. Bagged bunches were exposed to geraniol vapor emitted by the geraniol-treated polymer in the bags.

Geraniol, 3,7-dimethylocta-trans-2,6-dien-1-ol (Figure 1A), is an acyclic monoterpene alcohol that has a characteristic rose-like odor [21]. Geraniol has been identified in wines and contributes to wine aroma and flavor [22]. Although the diverse pharmacological activities of geraniol, including antitumor, anti-inflammatory, antioxidative, and antimicrobial activities, have been summarized in clinical applications [23], no studies have been carried out to investigate the effects of geraniol on anthocyanin accumulation in grape berry skin. Here, we report the positive effects of geraniol on the coloration of grape berry skin determined through a field experiment. We also demonstrate that geraniol-triggered anthocyanin accumulation in grape berry skin involves ABA membrane transport, not ABA biosynthesis.

## 2. Results

### 2.1. Effect of Geraniol Treatment on Anthocyanin Accumulation in Grape Berry Skins of Field-Grown Grapevines

Bunches of field-grown grapevines were exposed to 10 nM geraniol from véraison to harvest in the 2021 growing season. With regard to geraniol treatment of bunches of field-grown grapevines, each bunch was covered with a grape protective bag containing the geraniol-treated polymer at véraison (Figure 1C). The bunches were exposed to geraniol vapor emitted by the polymer in the bag from véraison to harvest (Figure 1D).

**Figure 2:**
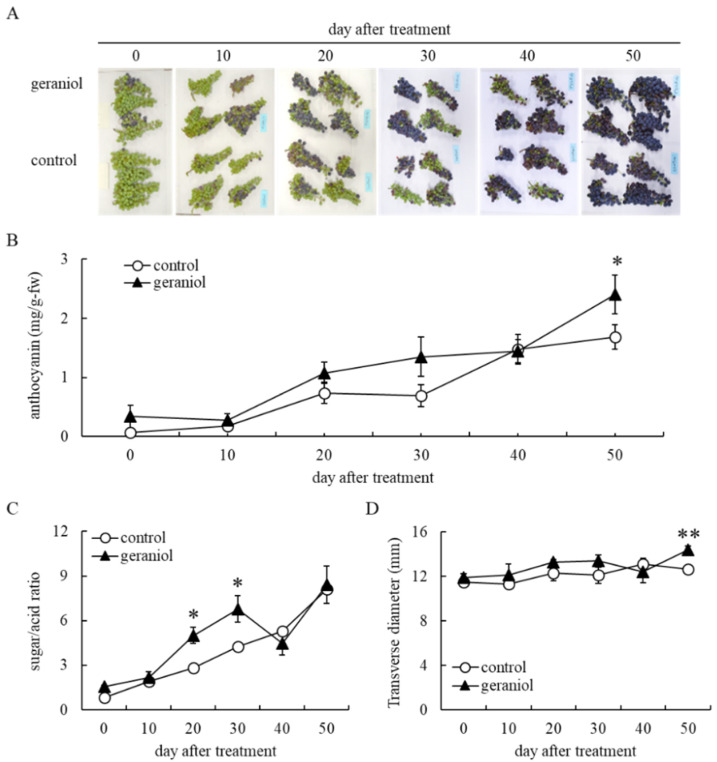
Effect of geraniol on anthocyanin accumulation in grape berry skins. Bunches of field-grown grapevines were treated with 10 nM geraniol as shown in Figure 1. (**A**) Photographs of bunches. (**B**) Anthocyanin contents in berry skins. (**C**) Sugar/acid ratios in juices. (**D**) Transverse diameters of berries. Data indicate means ± standard errors for four bunches. * *p* < 0.05, ** *p* < 0.01 compared with control. Geraniol, treated with 10 nM geraniol. Control, treated with diluted ethanol.

Geraniol-enhanced berry skin coloration was observed by the naked eye 20 days after treatment (Figure 2A). Although there was no significant difference, the geraniol-treated bunches tended to accumulate more anthocyanin in berry skins than the control bunches 20 and 30 days after treatment (Figure 2B). Although the effects of geraniol on anthocyanin accumulation in berry skins became negligible 40 days after treatment, the geraniol-treated bunches at harvest accumulated more anthocyanin in berry skins than the control bunches 50 days after treatment (Figure 2B). The sugar/acid ratio (Figure 2C) and the transverse diameter (Figure 2D) during berry maturation were comparable between geraniol-treated and control bunches, although significant differences were detected depending on the day after treatment.

Taken together, the field experiment suggested that treating bunches of field-grown grapevines with geraniol accelerated anthocyanin accumulation in berry skins.

### 2.2. Effect of Geraniol Treatment on the Transcription of Anthocyanin-Biosynthesis-Related Genes in Grape Berry Skins of Field-Grown Grapevines

**Figure 3:**
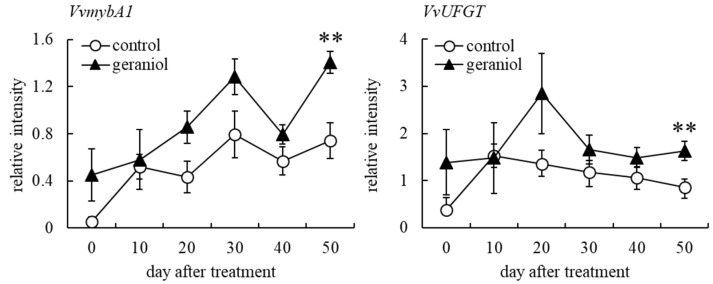
Effect of geraniol on transcription of *VvmybA1* and *VvUFGT* in grape berry skins. Bunches of field-grown grapevines were treated with 10 nM geraniol as shown in Figure 1. Transcription levels of *VvmybA1* (**left**) and *VvUFGT* (**right**) in berry skins were estimated by real-time RT-PCR. Data were calculated as gene expression relative to *β-actin* expression. Bars indicate means ± standard errors for four bunches. ** *p* < 0.01 compared with control. Geraniol, treated with 10 nM geraniol. Control, treated with diluted ethanol.

Anthocyanin accumulation in berry skin is determined by the expression of *mybA1*, which encodes a transcription factor for the expression of gene encoding UDP glucose flavonoid 3-*O*-glucosyl transferase (UFGT) that catalyzes anthocyanidin glycosylation, in grapevines [24]. Although no significant difference was detected, the transcripts of *VvmybA1* and *VvUFGT* in berry skins of geraniol-treated bunches were more abundant than those of control bunches 20, 30, and 40 days after treatment (Figure 3). Geraniol-treated bunches at harvest (50 days after treatment) showed a significant upregulation of *VvmybA1* and *VvUFGT* expression in berry skins compared with control bunches (Figure 3). The results suggested that the acceleration of anthocyanin accumulation in berry skins by geraniol occurred through the upregulation of *VvmybA1* transcription.

### 2.3. Effect of Geraniol Treatment on Anthocyanin Accumulation in Grape Cultured Cells

**Figure 4:**
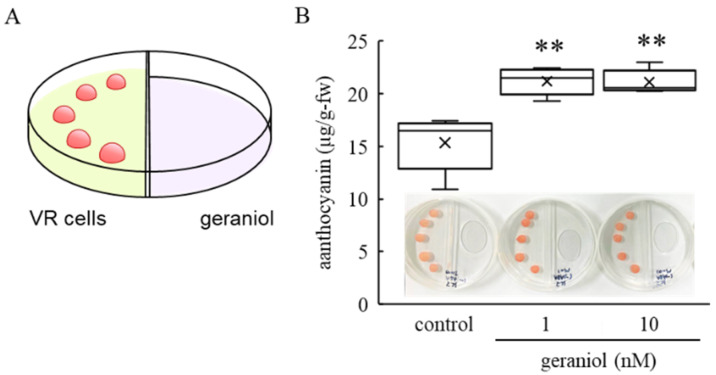
Effect of geraniol on anthocyanin accumulation in grape cultured cells. (**A**) Experimental design for VR cell treatment with geraniol. VR cells were incubated on modified LS agar medium in one section of a split Petri dish having two sections, and 1 or 10 nM geraniol or diluted ethanol as control solution was added into the other section of the same dish. (**B**) Anthocyanin content in VR cells treated with geraniol 10 days after treatment. Crosses (×) indicate means of five independent experiments. Representative VR cells are shown in the graph. ** *p* < 0.01 compared with control. Control, treated with diluted ethanol.

To determine the mechanisms of the enhanced anthocyanin accumulation triggered by geraniol, VR cells were used as a model for anthocyanin accumulation. Using a split Petri dish having two sections, we treated VR cells with geraniol (Figure 4A). Geraniol also enhanced anthocyanin accumulation in VR cells. VR cells treated with 1 and 10 nM geraniol accumulated more anthocyanin than control cells 10 days after treatment (Figure 4B).

### 2.4. Identification of Upregulated Genes by Geraniol Treatment Using Reverse Transcription-Polymerase Chain Reaction (RT-PCR)-Based Differential Display

**Figure 5:**
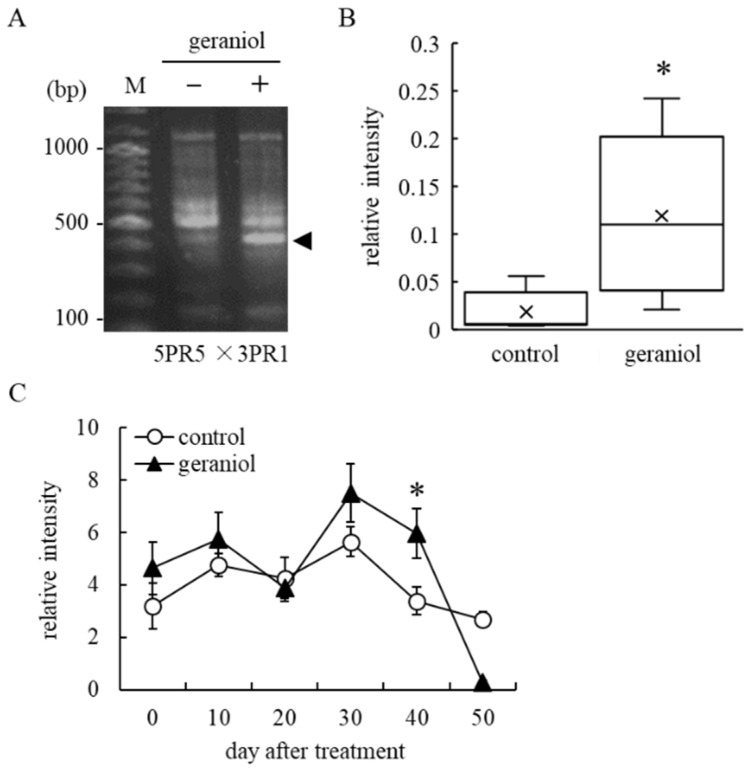
Upregulation of *VvABCG40* transcription by geraniol treatment in grape cultured cells and grape berry skins. (**A**) RT-PCR-based differential display. Upregulation of *VvABCG40* transcription (arrowhead) in response to geraniol was detected in geraniol-treated VR cells. M, 100 bp DNA ladder marker. +, geraniol treatment. –, no treatment. Numbers on the left indicate the positions of DNA molecular size markers. (**B**) Transcription levels of *VvABCG40* in VR cells. Transcription levels of *VvABCG40* in geraniol-treated or control cells were estimated by real-time RT-PCR. Data were calculated as gene expression relative to *β-actin* expression. Bars indicate means ± standard errors of five independent experiments. ×, means. (**C**) Transcription levels of *VvABCG40* in berry skins of field-grown grapevines. Transcription levels of *VvABCG40* in berry skins collected from geraniol-treated or control bunches of field-grown grapevines were estimated by real-time RT-PCR. Data were calculated as gene expression relative to *β-actin* expression. Bars indicate means ± standard errors for four bunches. * *p* < 0.05 compared with control. Geraniol, treated with 10 nM geraniol. Control, treated with diluted ethanol.

Cloning of genes that were upregulated by geraniol treatment was performed using RT-PCR-based differential display with 81 primer sets [25]. By comparing the profiles of the RT-PCR products amplified by each primer set between geraniol-treated and untreated VR cells, a geraniol-upregulated RT-PCR product was detected in the RT-PCR amplicons with 5PR5 (5′-AGTGGGATCA-3′) and 3PR1 (5′-TTTTTTTTTTTCC-3′) primer sets (Figure 5A). Analysis of the nucleotide sequence of the RT-PCR product by BLAST search revealed that it had 100% homology to *VvABCG40* (NM_001301124) encoding the ATP-binding cassette transporter G family protein (ABCG). RT-PCR analysis using *VvABCG40*-specific primers showed that *VvABCG40* transcripts were more abundant in the geraniol-treated VR cells than in the untreated cells (Figure 5B). The upregulation of *VvABCG40* transcription in response to geraniol was also observed in berry skins of field-grown grapevines. Geraniol-treated bunches showed upregulated *VvABCG40* transcription in berry skins 40 days after treatment compared with control bunches (Figure 5C). These results suggested that geraniol upregulated *VvABCG40* transcription in berry skins.

### 2.5. Geraniol Enhances Transcription of ABA-Responsible Gene, but Not ABA-Biosynthesis-Related Gene, in Grape Berry Skins of Field-Grown Grapevines

**Figure 6:**
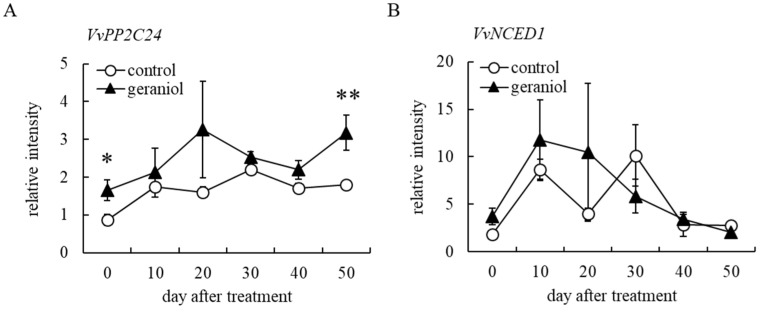
Effect of geraniol on *VvPP2C24* and *VvNCED1* transcription in grape berry skins. (**A**) Transcription levels of *VvPP2C24* in berry skins. (**B**) Transcription levels of *VvNCED1* in berry skins. The transcription levels in berry skins collected from geraniol-treated or control bunches of field-grown grapevines were estimated by real-time RT-PCR. Data were calculated as gene expression relative to *β-actin* expression. Bars indicate means ± standard errors for four bunches. * *p* < 0.05, ** *p* < 0.01 compared with control. Geraniol, treated with 10 nM geraniol. Control, treated with diluted ethanol.

VvABCG40 is a grapevine ortholog of the *Arabidopsis* ABC transporter G family protein and functions as an ABA membrane transporter [26,27]. To evaluate the effects of geraniol on ABA biosynthesis, the transcription of *VvPP2C24*, which encodes ABA-responsible type 2C protein phosphatases [28] and *VvNCED1*, which encodes a key enzyme in ABA biosynthesis [29] was evaluated in berry skins of field-grown grapevines. Geraniol-treated bunches showed a significant upregulation of *VvPP2C24* expression in berry skins at harvest (50 days after treatment) compared with control bunches (Figure 6A). In contrast, geraniol did not increase *VvNCED1* transcription levels in berry skins during berry maturation (Figure 6B).

## 3. Discussion

**Figure 7:**
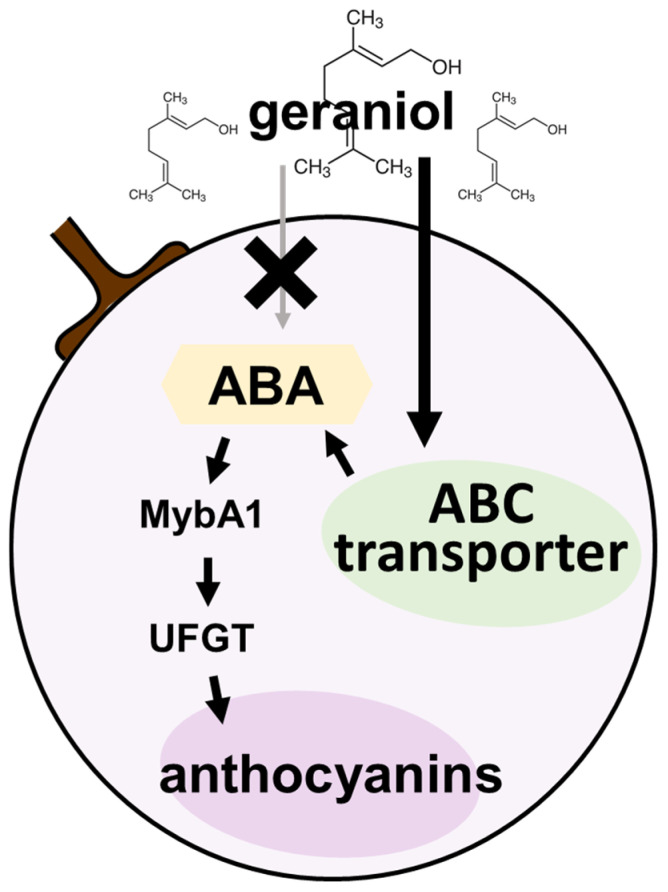
Predicted pathway for geraniol-induced anthocyanin accumulation in grape berry skin.

A predicted pathway for geraniol-induced anthocyanin accumulation in grape berry skin is shown in Figure 7. Treatment of bunches of field-grown grapevines at véraison with geraniol resulted in the upregulation of the transcription of *VvABCG40*, a gene that encodes ABC transporter and functions as an ABA membrane transporter [26]. Plant ABCG40 catalyzed ATP-dependent ABA uptake by target tissues [30]. The increase of endogenous bioactive ABA from véraison accelerates anthocyanin accumulation in grape berry skin [14]. In general, the enhancement of ABA-induced anthocyanin accumulation in grape berry skin by the exogenous application of chemical stimulants is caused by the activation of ABA biosynthesis [15], the release of bioactive ABA from glucose-conjugated ABA [16] and/or ABA membrane transport [26]. Vanillylacetone activated ABA production in grape berry skin [15], whereas allantoin increased the production of bioactive ABA through the β-glucosidase-catalyzed hydrolysis of glucose-conjugated ABA but not through the ABA biosynthesis [16]. Enhancement of *VvABCG40* transcription was observed in deficit-irrigation-driven anthocyanin accumulation in grape berry skin [31]. The retardation of anthocyanin accumulation in grape berry skin by the application of synthetic strigolactone analog GR24 was accompanied by the downregulation of *VvABCG40* transcription [27]. Thus, VvABCG40-driven ABA membrane transport has a strong relationship with anthocyanin accumulation in grape berry skin. In the present study, we identified *VvABCG40* as an upregulated gene by geraniol treatment. Geraniol upregulated *VvABCG40* transcription in both grape cultured cells and berry skins. In contrast, the geraniol-triggered transcription of *VvNCED1*, which encodes a key enzyme in ABA biosynthesis, was not detected in berry skins. Genome-wide identification of the grape *NCED* gene family found a total of 12 *NCED* genes in the grape genome [32]. ABA concentration in grape berry was highly correlated with *VvNCED1* transcript abundance [33]. Although we could not evaluate the activity of ABA transport by VvABCG40 due to technical problems, the increase of ABA content in berry skins treated with geraniol was indirectly supported by the finding that geraniol upregulated the transcription of ABA-responsible *VvPP2C24* in the skins. Taken together, geraniol might enhance anthocyanin accumulation in berry skins through the upregulation of *VvmybA1* or *VvUFGT* transcription by VvABCG40-driven ABA membrane transport.

How is geraniol recognized by grape cells? In human-derived enteroendocrine cells, geraniol is recognized by the intestinal olfactory-type G protein receptor, which leads to the secretion of glucagon-like peptide-1 [34]. It was reported that geraniol at a high concentration interacts with human estrogen receptor in recombinant yeast cells [35]. It was also demonstrated that geraniol has the potential to penetrate human skin [36]. Geraniol directly perturbs the function of the plasma membrane by inducing membrane depolarization in human colon cancer cells, which results in the suppression of cell proliferation [37]. Similarly, an irreversible loss of cell membrane integrity in geraniol-treated grape cultured cells was observed [38]. Although the recognition mechanisms of geraniol in plant cells have yet to be defined, we found that geraniol was recognized by multiple types of grape cells, which led to the enhancement of anthocyanin accumulation in grape cells. Further investigation of the early events in the geraniol–plant cell interaction may provide new information on the function of geraniol in plant physiological phenomena including plant–plant communication.

For the past several decades, the average temperatures in wine-grape-producing regions around the world have continued to increase, culminating in loss of berry quality including anthocyanin content in grape berry skin [39,40]. To promote anthocyanin accumulation in grape berry skin under future climate conditions, many researchers have explored specific viticultural practices [7,8,9,11] and chemical applications [13,15,16,41,42,43]. Interestingly, the simultaneous increase of anthocyanin and geraniol contents in grape berries subjected to viticultural practices or chemical application was occasionally observed. For example, deficit irrigation of Merlot wine grapes increased both anthocyanin and geraniol contents in berries [44]. Postharvest treatment of Aleatico wine grape bunches with ethylene increased anthocyanin content and induced an increase of geraniol content in berries [45]. In the present study, we were unable to confirm whether exogenous geraniol treatment affects endogenous geraniol content in grape berries. Geraniol biosynthesis is mainly restricted to grape berry skin [46], and 1-deoxy-d-xylulose 5-phosphate (DOXP) is used as a precursor in the plastidial DOXP/2-C-methyl-d-erythritol-4-phosphate (MEP) pathway [47]. Although whether endogenous geraniol stimulates anthocyanin accumulation in grape berry skins remains an unresolved issue, we found that treatment to bunches of field-grown grapevines with geraniol had the potential to improve anthocyanin content in berry skins at harvest. Geraniol treatment affected neither the vegetative nor the reproductive growth of grapevines evaluated during the growing season (data not shown). Further field experiments are necessary to clarify the best conditions for geraniol treatment, including the dosage of geraniol and the timing of treatment, and to elucidate whether grape berries treated with geraniol contribute to wine color and aroma.

## 4. Materials and Methods

### 4.1. Plant Materials

*Vitis vinifera* cv. Cabernet Sauvignon was cultivated in the experimental vineyard of The Institute of Enology and Viticulture, University of Yamanashi, Japan (latitude 35.6800524, longitude 138.569268, elevation 250 m). The grapevines were approximately 30 years old and trained in the Guyot-style training system. 

Anthocyanin-producing VR cells, which are grape cultured cells derived from the anthers of *Vitis* interspecific hybrid cultivar Bailey Alicante A [48], were obtained from the RIKEN BioResource Center (sample no. rpc00003). VR cells were maintained on a modified Linsmaier and Skoog (LS) agar medium (pH 5.7) supplemented with 3% sucrose, 0.2 mg/L kinetin, and 0.05 mg/mL 2,4-dichlorophenoxyacetic acid.

### 4.2. Geraniol Treatment of Field-Grown Grape Bunches

Bunches of six field-grown grapevines were prepared for geraniol treatment in the 2021 growing season. Geraniol (1.54 g, Fuji Film Wako Pure Chemical, Osaka, Japan) was dissolved in 10 mL of ethanol (99.5%, Fuji Film Wako Pure Chemical), and 1 M geraniol was obtained as a stock solution. Then, a 10 nM geraniol solution was prepared by diluting with sterilized water. As a control solution, Milli Q (1.54 g) was added to 10 mL of ethanol, and the solution was diluted 1000 times with sterilized water. Six grams of a water-absorbing polymer, sodium polyacrylate (2 mm in diameter, Artec, Osaka, Japan, Figure 1B), was soaked in 400 mL of 10 nM geraniol or control solution at room temperature overnight. Eighteen grams of polymer were added into a grape protective bag (21.5 × 31.5 cm, Kobayashi Bag Mfg., Nagano, Japan) having both translucency and air-permeability, and each bunch at véraison was covered with the bag (4 August 2021, Figure 1C). Four bunches were randomly collected every 10 days from véraison to harvest (50 days after treatment).

### 4.3. Geraniol Treatment of VR Cells

VR cells were incubated on the modified LS agar medium at 27 °C in the dark for 7 d. For geraniol treatment, a split Petri dish with two sections (φ 90 × 15 mm, Sansei Medical, Kyoto, Japan) was used. VR cells were incubated on 10 mL of modified LS agar medium in one section, and 5 mL each of 1 and 10 nM geraniol or control solution was added into the other section of the same dish. After incubation for 10 days at 27 °C under light irradiation (96.2 µmol·m^−2^·s^−1^/16 h/d), the cells were subjected to anthocyanin measurement and gene expression analysis.

### 4.4. Berry Characteristics

Twenty grape berries (six from the top, eight from the middle, and six from the bottom) were collected from each bunch and hand-pressed to obtain juice. Soluble solid content (Brix) and tartaric acid level (g/100 mL) in the juices were measured with a refractometer (PAL-BX/ACID2, Atago, Tokyo, Japan). The soluble solid content/tartaric acid ratio (sugar/acid ratio) was calculated.

The transverse diameters of the twenty grape berries collected from each bunch were measured using a digital caliper (CDN-100, Niigata Seiki Co., Ltd., Niigata, Japan).

### 4.5. Total Anthocyanin Measurement

For the measurement of anthocyanin in berry skins, twenty berries (six from the top, eight from the middle, and six from a bottom) were collected from each bunch. Skins were peeled off from the berries using tweezers and frozen immediately in liquid nitrogen. 

Anthocyanin extraction from VR cells and berry skins and its measurement were performed as described previously [49] with modification. Briefly, VR cells or berry skins were placed in a mortar containing liquid nitrogen and homogenized with a pestle. Approximately 1 g of the pulverized sample was macerated in 10 mL of HCl-methanol [36:1 (*v*/*v*)] at 4 °C in the dark overnight. After mixing, the mixture was centrifuged at 11,000× *g* for VR cells or at 3000× *g* for berry skins at room temperature for 5 min, respectively. The absorbance (OD_520_) of the supernatants was measured using a spectrometer (ASV11D-H, AS ONE, Osaka, Japan). Total anthocyanin content was calculated using a previously published formula [49] and converted into malvidin-3-glucoside equivalent as µg or mg per gram of fresh weight of VR cells or fresh skin weight.

### 4.6. RNA Isolation

VR cells or berry skins were placed in a mortar containing liquid nitrogen and homogenized with a pestle. The samples were further crushed using TissueLyser II (Qiagen, Hilden, Germany) according to the manufacturer’s instructions. Total RNA isolation from the pulverized samples was performed using a Fruit-mate for RNA Purification (Takara Bio, Shiga, Japan) and an RNeasy Plant Mini Kit (Qiagen) with QIAcube (Qiagen) according to the manufacturer’s instructions.

### 4.7. RT-PCR-Based Differential Display

To identify the genes that were upregulated in response to geraniol, RT-PCR-based differential display was performed according to the method described previously [25]. Briefly, total RNA isolated from geraniol-treated VR cells or control was reverse-transcribed using oligo(dT) primer and PrimeScript RT Reagent Kit (Perfect Real Time) (Takara Bio). PCR amplification was performed using both 3′ and 5′ arbitrary primers [25] for 25 cycles at 95 °C for 20 s, 60 °C for 30 s, and 72 °C for 30 s, with a final extension step at 72 °C for 5 min. The RT-PCR products were subjected to electrophoresis using 2% agarose gel and visualized by ethidium bromide staining under UV illumination. The differentially expressed products were extracted from the agarose gel using a NucleoSpin Gel and PCR Clean-up Kit (Takara Bio) and cloned into a pMD20-T vector (Takara Bio). Nucleotide sequences were confirmed by DNA sequencing (Fasmac, Kanagawa, Japan) and analyzed by NCBI BLAST search.

### 4.8. Real-Time RT-PCR

cDNA was synthesized from total RNA using a PrimeScript RT Reagent Kit with gDNA Eraser (for Perfect Real Time) (Takara Bio) according to the manufacturer’s instructions. Real-time RT-PCR was performed with TB Green Premix Ex Taq II (Tli RNaseH Plus) (Takara Bio). PCR amplification was performed for 40 cycles at 95 °C for 5 s and at 60 °C for 30 s and one cycle at 95 °C for 15 s and at 60 °C for 30 s after an initial denaturation at 95 °C for 30 s. The primers used for PCR amplification are shown in Supplementary Table S1. The dissociation curves were evaluated to verify the specificity of the amplification reaction. The expression levels of each gene were determined as the number of amplification cycles needed to reach a fixed threshold by the standard curve method with Thermal Cycler Dice Real-Time System Single Software ver. 3.00 (Takara Bio). Data are expressed as relative intensities to β-actin used for data normalization.

### 4.9. Statistical Analysis

Data calculated from biological replicates are shown as means ± errors in line graphs or box plots. Statistical analysis was performed by Dunnett’s multiple comparison test or the Student’s *t*-test using Excel statistics software 2012 (Social Survey Research Information, Tokyo, Japan).

## Data Availability

Not applicable.

## Supplementary Materials

The following supporting information can be downloaded at: https://www.mdpi.com/article/10.3390/plants11131694/s1, Supplementary Table S1: Sequences of primers for real-time RT-PCR.
