# Supplementary material for: Geraniol as a Potential Stimulant for Improving Anthocyanin Accumulation in Grape Berry Skin through ABA Membrane Transport
Source: Plants (Basel). 2022 Jun 27;11(13):1694. doi: 10.3390/plants11131694 (PMC9269297; doi:10.3390/plants11131694)
Supplement: Supplementary file 1 [file plants-11-01694-s001.zip › plants-1767345-supplementary.pdf]

Supplementary Table S1 Sequences of primers for real-time RT-PCR

Name	Sequence	GenBank accession
VvmybA1 (forward)	5'-ATCCCAGAAGCCCACATCAA-3'	AB111101
VvmybA1 (reverse)	5'-GCAAGCCTCAGGACAGAAGAA-3'	AB111101
VvUFGT[a] (forward)	5'-CTTCTTCAGCACCAGCCAATC-3'	AB047099
VvUFGT (reverse)	5'-AGGCACACCGTCGGAGATAT-3'	AB047099
VvABCG40[b] (forward)	5'-AGCCCTTCTTCACCCAATTT-3'	NM_001301124
VvABCG40 (reverse)	5'-CCCAAGGAAGAGAACAGCAG-3'	NM_001301124
VvNCED1[c] (forward)	5'-GAGACCCCAACTCTGGCAGG-3'	AY337613
VvNCED1 (reverse)	5'-AAGGTGCCGTGGAATCCATAG-3'	AY337613
VvPP2C24[d] (forward)	5'-TTAAAGCCCTTCGTGAGCTG-3'	XM_002282572
VvPP2C24 (reverse)	5'-GACACCACGTCCCACAGAC-3'	XM_002282572
β-actin (forward)	5'-CAAGAGCTGGAAACTGCAAAGA-3'	AF369524
β-actin (reverse)	5'-AATGAGAGATGGCTGGAAGAGG-3'	AF369524

[a]UFGT, UDP glucose flavonoid 3-*O*-glucosyl transferase

[b]ABCG40, ABC transporter G family protein 40

[c]NCED1, 9-cis-epoxycarotenoid dioxygenase 1

[d]PP2C24, highly ABA-induced type 2Cprotein phosphatase 24
